# Supplementary material for: The neural basis for a persistent internal state in Drosophila females
Source: eLife. 2020 Nov 23;9:e59502. doi: 10.7554/eLife.59502 (PMC7787663; doi:10.7554/eLife.59502)
Supplement: Transparent reporting form [file elife-59502-transrepform.docx]

***eLife’s* transparent reporting form**

We encourage authors to provide detailed information *within their submission* to facilitate the interpretation and replication of experiments. Authors can upload supporting documentation to indicate the use of appropriate reporting guidelines for health-related research (see [EQUATOR Network](http://www.equator-network.org/%20)), life science research (see the [BioSharing Information Resource](https://biosharing.org/)), or the [ARRIVE guidelines](http://www.plosbiology.org/article/info:doi/10.1371/journal.pbio.1000412) for reporting work involving animal research. Where applicable, authors should refer to any relevant reporting standards documents in this form.

If you have any questions, please consult our Journal Policies and/or contact us: [editorial@elifesciences.org](mailto:editorial@elifesciences.org).

**Sample-size estimation**

- You should state whether an appropriate sample size was computed when the study was being designed
- You should state the statistical method of sample size computation and any required assumptions
- If no explicit power analysis was used, you should describe how you decided what sample (replicate) size (number) to use

Please outline where this information can be found within the submission (e.g., sections or figure legends), or explain why this information doesn’t apply to your submission:

No explicit power analysis was used, and the number of flies per experimental condition was chosen based on previous experiments similar experiments conducted in our lab. The number of flies used and the statistical tests are detailed in the figure legends. General details on the statistical methods used, and details about specific analyses done where details beyond those provided in the figure legends are required, could be found under Materials and Methods/Statistical analysis.

**Replicates**

- You should report how often each experiment was performed
- You should include a definition of biological versus technical replication
- The data obtained should be provided and sufficient information should be provided to indicate the number of independent biological and/or technical replicates
- If you encountered any outliers, you should describe how these were handled
- Criteria for exclusion/inclusion of data should be clearly stated
- High-throughput sequence data should be uploaded before submission, with a private link for reviewers provided (these are available from both GEO and ArrayExpress)

Please outline where this information can be found within the submission (e.g., sections or figure legends), or explain why this information doesn’t apply to your submission:

For all experiments data acquisition was intermingles with the between controls experimentals, and we aimed for equal number of controls and experimentals. The minimal number of flies per experiment where predefined before the experiments, based on previous experience. For the behavioral experiments we used a minimum of 30 flies, except it the 2-minute and 30-seconds activation conditions where we used a minimum of 20 individuals per condition. In some cases we used more flies than minimum, but in no case we added flies after analyzing the data. We did not sort out outliers in any experiment reported in this study. For the behavioral data, in a few cases (<10% of the flies for any experiment) we could not use all the flies for the analysis of the behavior because of technical issues such as synchronization between video and audio. These flies were discarded from the analysis, before any behavioral analysis was done, and therefore did not cause any bias. We used multiple repetitions of clustering when analyzing the behavioral data (Figure 1A-D and figure 1 – figure supplement 2A-C). This procedure is detailed under Materials and Methods and in the figure legends, where we describe in details the statistical analysis.

**Statistical reporting**

- Statistical analysis methods should be described and justified
- Raw data should be presented in figures whenever informative to do so (typically when N per group is less than 10)
- For each experiment, you should identify the statistical tests used, exact values of N, definitions of center, methods of multiple test correction, and dispersion and precision measures (e.g., mean, median, SD, SEM, confidence intervals; and, for the major substantive results, a measure of effect size (e.g., Pearson's r, Cohen's d)
- Report exact p-values wherever possible alongside the summary statistics and 95% confidence intervals. These should be reported for all key questions and not only when the p-value is less than 0.05.

Please outline where this information can be found within the submission (e.g., sections or figure legends), or explain why this information doesn’t apply to your submission:

We reported the number of flies used for each analysis, the statistical methods and the significance in the figure legends. Additional details are mentioned under Materials and Methods/Statistical analysis.

(For large datasets, or papers with a very large number of statistical tests, you may upload a single table file with tests, Ns, etc., with reference to sections in the manuscript.)

**Group allocation**

- Indicate how samples were allocated into experimental groups (in the case of clinical studies, please specify allocation to treatment method); if randomization was used, please also state if restricted randomization was applied
- Indicate if masking was used during group allocation, data collection and/or data analysis

Please outline where this information can be found within the submission (e.g., sections or figure legends), or explain why this information doesn’t apply to your submission:

The description of each experimental group could be find in the text, as well as in the Key Resources Table (where the genotype used for each experiment is detailed). The number of individuals in each group is detailed in the figure legend. In cases where statistics was based on multiple repetitions of clustering (Figure 1A-D and figure 1 – figure supplement 2A-C), the details are and justification for the statistical analysis are in the Materials and Methods section.

**Additional data files (“source data”)**

- We encourage you to upload relevant additional data files, such as numerical data that are represented as a graph in a figure, or as a summary table
- Where provided, these should be in the most useful format, and they can be uploaded as “Source data” files linked to a main figure or table
- Include model definition files including the full list of parameters used
- Include code used for data analysis (e.g., R, MatLab)
- Avoid stating that data files are “available upon request”

Please indicate the figures or tables for which source data files have been provided:

We listed all the cells used in this paper connectome analysis (Table 1), including the coordinates of each cell in Flywire (Table 1). The major tool used for behavioral tracking is ‘sleap’ (<https://sleap.ai/>) that was developed in our lab and is available online.
